# Can we really predict the respiratory morbidity of preterm birth?

**DOI:** 10.1038/s41390-025-04012-1

**Published:** 2025-03-18

**Authors:** Avinash Kondiboyina, Samuel B. Axford, David G. Tingay

**Affiliations:** 1 Neonatal Research, Murdoch Children’s Research Institute, Parkville, VIC Australia; 2 Department of Neonatal Medicine, The Royal Children’s Hospital, Parkville, VIC Australia; 3 Department of Paediatrics, University of Melbourne, Melbourne, VIC Australia; 4 Department of Critical Care, University of Melbourne, Melbourne, VIC Australia

*‘We know what we are, but not what we may be?’* Shakespeare (Hamlet Act 4 Scene 5)

Despite advances in treatments, long-term respiratory morbidity remains a major consequence of preterm birth, with rates of bronchopulmonary dysplasia (BPD) unchanged.^1^ BPD is the result of a cascade of lung injury-inflammation events throughout the antenatal and postnatal course, particularly in the first few days after birth.^1^ Thus, applying effective treatments early is essential to reduce BPD rates. However, this has been difficult to achieve; the progression to BPD is not linear and knowing when to offer a specific treatment is often hampered by a lack of reliable descriptors of BPD risk.^2^ Furthermore, many treatments carry risks; corticosteroids are potent anti-inflammatory agents that reduce BPD rates but increase the risk of neurological complications and should be reserved for infants with the greatest BPD risk.^3^ Thus, clinicians need better ways to stratify preterm infants based on both treatment risk and effects.

In this edition of *Pediatric Research*, Kanzawa and colleagues report a simplified model to predict severe BPD (defined as need for supplementary oxygen at discharge to home or death due to BPD) in preterm infants.^4^ To train the model, they included data from 2026 preterm infants from 21 centers in Aichi Prefecture (Japan) born at <32 weeks gestational age (GA) or <1500 g birth weight and who received ventilation for at least 7 days after birth. To develop a simple and clinically useful model, they used classification and regression tree (CART) techniques using only 2 predictors: respiratory severity (RS) scores at 7 days and 14 days after birth, and GA. The resulting models for two timepoints (“Postnatal Day 7” and “Postnatal Day 14”) demonstrated good C-statistics of 0.789 (Day 7) and 0.779 (Day 14). The authors also used an external validation dataset of 387 preterm infants from Saitama Prefecture (Japan) and showed good calibration plots and C-statistics of 0.753 (Day 7) and 0.827 (Day 14).

Models to predict BPD are not new.^5,6^ Published models have incorporated multiple predictors that are either not readily available or too general to describe the nuance of preterm lung disease.^7^ The role of GA, an indicator of the developmental immaturity of the lung, logically confers the greatest signal for risk. Clinically, a model that predicts an infant born at 23 weeks’ GA is at higher risk for BPD than one born at 28 weeks’ GA is of little practical benefit. Rather, clinicians need to know an individual risk profile for a baby born at a specific GA. The proposed model of Kanzawa and colleagues seeks to find a pragmatic balance, relying on only two variables derived from readily available non-invasive clinical data, RS score and GA, making it easy to collect and highly practical for routine neonatal care. The RS score incorporates oxygen needs and mean airway pressure. Mean airway pressure is a reliable marker of the diseased lung’s physiological state that is independent of the mode of respiratory support and allows weighting of risk at any GA.^8^

CART constructs decision trees through a binary splitting method. The tree starts at a “root node”, which contains the entire dataset. A predictor and its threshold value are chosen that best splits the dataset into 2 groups or “nodes” that are as “pure” as possible. The theoretical maximum purity would be a perfect split of severe BPD and no severe BPD outcomes, but there is always some “impurity”. This process is repeated at each node, creating a tree structure with the aim of reducing the impurity, until a specific criterion is reached. At this point, the resulting terminal nodes (“leaves”), represent the outcomes of the classification task (severe BPD and no severe BPD). This is akin to playing “20 Questions”. Each question helps narrow down the possibilities, making the final decision more accurate. Some drawbacks of CART models should be considered. The models tend to overfit the training data; however, the authors have protected against overfitting by including only two predictors and using cost-complexity pruning to remove branches that add little predictive value. Furthermore, even small changes in data lead to a completely different tree structure, making them unstable. The impurity optimisation occurs only locally at each split, which may not always lead to globally optimal trees. Random forest models address these drawbacks by generating random subsets of the dataset and predictors and creating a decision tree for each subset. It then combines the trees to create a more stable ensemble model that is robust to overfitting and small changes in the data and provides more accurate results. However, random forests are less interpretable owing to their ensemble nature. CART models on the other hand are highly interpretable because they are simple and clearly show their logic, which were the primary aims of the authors.

Critical to all prediction models is the choice of covariates used to define risk. The authors used univariate analysis to determine feature importance based on *p* value cutoffs. This method is simple, interpretable, and useful to understand relationships between individual predictors and the outcome. However, it ignores interactions between variables and is sensitive to sample size, with larger sample sizes making weak relationships appear significant. A random forest method considers feature interactions, is robust to noise and outliers, and captures non-linear and complex relationships among predictors; however, it ranks features only by relative importance and does not provide statistical significance. Both methods can be combined for more robust feature selection: random forest can robustly rank features by relative importance and univariate analysis can verify their statistical significance.^9^

Online calculators that require clinicians to input parameters are appealing, but the models are often a “black box”.^7^ In contrast, Kanzawa and colleagues’ model is a simple decision tree flowchart. This simplicity enhances clinical utility by reducing the cognitive and operational burden on busy clinicians and increases transparency in the risk calculation. The authors also present calibration curves to show the performance of the model at different BPD risk levels, which is absent in most other BPD prediction studies. Importantly, their study used population datasets and incorporated external validation from a different Japanese healthcare region, which many prior models lack.^6^ This is critical to demonstrating the model’s generalizability and robustness. Many BPD prediction models have poor generalizability when applied to different and often larger populations.^5,6^ It will be important to understand how the proposed model performs outside of the Japanese population and healthcare setting. The transparency and simplicity of the model should make this relatively easy. Of course, lack of precision in different populations does not exclude use but rather informs in which populations it can be applied.

An important distinction in this study is the definition of BPD. The authors chose to predict severe BPD, defined as death due to BPD or discharge to home requiring oxygen therapy; this is different from commonly used definitions.^1,10^ Comparisons with other BPD prediction models should be applied with caution. The model is not designed to predict ‘mild’ or even ‘any’ BPD. This is not unreasonable. Parents place more importance on the need for oxygen at discharge, rather than the diagnosis of BPD.^11^ The authors also highlight that predicting severe BPD rather than any BPD is likely to be of greater clinical use for risk stratification of treatment choices.

BPD rates have remained high in part due to the simplistic assumption that BPD is a single condition that can be treated with a single intervention. Rather, BPD is a method of categorising an ongoing heterogenous pattern of lung disease throughout perinatal life caused by a multitude of different processes.^1^ In this context, the value of prediction models is evident: Whether to guide population stratification for a trial or to guide clinicians in understanding the risks associated with a specific infant’s preterm journey. This also explains why prediction models become more accurate the older a preterm infant is (and closer to time of BPD diagnosis).^5,6^ The more events that occur, and the lower risk of new unexpected events, the more accurate the link to BPD. Importantly, prediction is not the solution to precision medicine. Prediction simply aims to allocate the risk of an adverse event to an individual. Alone it cannot inform specific treatment options in a heterogenous population. This requires first categorising the population based on their features or sub-phenotypes. Phenotyping early acute respiratory failure in adults improved selection of candidates for different critical care treatments^12^ and improved BPD prediction models in a pilot study in preterm infants (currently being investigated in a large cohort study ACTRN1262400085156).^13^

The phrase ‘*all models are wrong, but some are useful*’ coined by the statistician George Box is apt for BPD.^14^ No prediction model will be perfect; but considering the variable trajectory of respiratory disease following preterm birth, well-designed models that are applied to the right population at the right time are likely to be better than none.
